# The analysis of the skeletal muscle metabolism is crucial for designing optimal exercise paradigms in type 2 diabetes mellitus

**DOI:** 10.1186/s10020-024-00850-7

**Published:** 2024-06-10

**Authors:** Elias Abi Akar, Laure Weill, Mirella El Khoury, Cédric Caradeuc, Gildas Bertho, Suzan Boutary, Cynthia Bezier, Zoé Clerc, Delphine Sapaly, Sabrina Bendris, Flore Cheguillaume, Nicolas Giraud, Assaad A. Eid, Frédéric Charbonnier, Olivier Biondi

**Affiliations:** 1 Faculty of Basic and Biomedical Sciences, Université Paris Cité & Inserm UMR_S1124, 45 rue des Saints-Pères, 75270 Paris Cedex 06, France; 2 Department of Anatomy, Cell Biology and Physiological Sciences, Faculty of Medicine and Medical Center, American University of Beirut, Bliss Street, 11-0236, Riad El-Solh, Beirut, 1107-2020 Lebanon; 3 Faculty of Basic and Biomedical Sciences, Université Paris Cité & UMR8601 CNRS, 45 rue des Saints-Pères, 75270 Paris Cedex 06, France; 4 Inserm U1195, Bâtiment Gregory Pincus, 80 rue du Général Leclerc, 94276 Le Kremlin Bicêtre, France; 5 Laboratoire de Biologie de l’Exercice Pour la Performance et la Santé (LBEPS), UMR, Université d’Evry, IRBA, Université de Paris Saclay, 91025 Evry-Courcouronnes, France

**Keywords:** Type 2 diabetes mellitus, Mouse models, Precision exercise, Muscle metabolism

## Abstract

**Background:**

Type 2 diabetes mellitus (T2DM) is a chronic metabolic disease that commonly results from a high-calorie diet and sedentary lifestyle, leading to insulin resistance and glucose homeostasis perturbation. Physical activity is recommended as one first-line treatment in T2DM, but it leads to contrasted results. We hypothesized that, instead of applying standard exercise protocols, the prescription of personalized exercise programs specifically designed to reverse the potential metabolic alterations in skeletal muscle could result in better results.

**Methods:**

To test this hypothesis, we drew the metabolic signature of the fast-twitch quadriceps muscle, based on a combined unbiased NMR spectroscopy and RT-qPCR study, in several T2DM mouse models of different genetic background (129S1/SvImJ, C57Bl/6J), sex and aetiology (high-fat diet (HFD) or HFD/Streptozotocin (STZ) induction or transgenic MKR (FVB-Tg Ckm-IGF1R*K1003R)1Dlr/J) mice. Three selected mouse models with unique muscular metabolic signatures were submitted to three different swimming-based programs, designed to address each metabolic specificity.

**Results:**

We found that depending on the genetic background, the sex, and the mode of T2DM induction, specific muscular adaptations occurred, including depressed glycolysis associated with elevated PDK4 expression, shift to β-oxidation, or deregulation of amino-acid homeostasis. Interestingly, dedicated swimming-based exercises designed to restore specific metabolic alterations in muscle were found optimal in improving systemic T2DM hallmarks, including a significant reduction in insulin resistance, the improvement of glucose homeostasis, and a delay in sensorimotor function alterations.

**Conclusion:**

The muscle metabolism constitutes an important clue for the design of precision exercises with potential clinical implications for T2DM patients.

**Supplementary Information:**

The online version contains supplementary material available at 10.1186/s10020-024-00850-7.

## Introduction

With around 451 million adults affected worldwide in 2017, diabetes is one of the main global wellbeing concerns. Finding a cure for diabetes is highly challenging, notably due to its high heterogeneity in causes and consequences. Among all affections associated to diabetes, type 2 diabetes mellitus (T2DM), which accounts for 90% of overall diabetic cases, is defined as a co-morbidity factor due to the involvement of numerous organs leading to non-alcoholic fatty liver disease (Saponaro et al. 2015), cardiovascular alterations (Gu et al. 1999), retinopathy (Fong et al. 2004), nephropathy (Vigersky 2011), or neuropathy (Zatalia and Sanusi 2013). The development of T2DM is gradual and mainly related to glucose toxicity due to insulin resistance and/or abnormal insulin secretion from pancreatic β-cells (Abdul-Ghani and DeFronzo 2009), leading to a wide variety of subtypes (Elksnis et al. 2019). Moreover, T2DM patients generally display an excessive concentration of circulating free fatty acids (FAs), mainly due to decreased ability of the liver to metabolize Fas (Firneisz 2014), which worsens the insulin resistance (Sears and Perry 2015). Physical activity is recommended as one first-line treatment in T2DM adults, but with widely varying even contrasting results non-resolved yet (Honkola et al. 1997; Dunstan et al. 2002, 2005; Castaneda et al. 2002; Kadoglou et al. 2012; Balducci et al. 2012; Krause et al. 2014; Amanat et al. 2020). An important aspect still relatively unexplored in T2DM that could greatly influence patient response to exercise is the muscle metabolic status which could vary depending on the individual pathological patterns (Maier and Gould 2000; DeFronzo and Tripathy 2009; Tumova et al. 2016). Muscle is indeed crucially involved in the modulation of whole-body metabolism as it constitutes the largest metabolic organ and accounts for 30% of the basal metabolic rate (Zurlo et al. 1990). It remains the principal consumer of glucose during exercise and of lipids for post-exercise recovery (Hargreaves and Spriet 2020). Importantly, skeletal muscle is responsible for the major part (> 80%) of insulin-stimulated whole-body glucose uptake and hence plays an important role in insulin resistance (Phielix and Mensink 2008). Undoubtedly, submitting T2DM patients to a standard exercise protocol, regardless of the pathophysiological characteristics of their diabetes notably at the level of the muscle metabolism, and the inter-individual specificities is unlikely to produce optimal benefits. Thus, our hypothesis is that personalized exercise protocols, aimed at improving muscle metabolism in a specific manner for each patient, as it is done in precision medicine, could induce important benefits in terms of symptoms and quality of life.

To test this hypothesis, we submitted different mouse models of T2DM, either induced by high-fat diet (HFD), a combination of HFD and pancreatic toxin Streptozotocin (STZ) injection or genetically (Ckm-IGF1R*K1003R)1Dlr/J), to different exercise protocols based on swimming. The exercise protocol was designed to target the muscle metabolic pathways that are differentially altered in the fast-twitch quadriceps muscle of the different T2DM mouse populations. We found that regardless of the identity of the altered energy metabolism pathway, which depends on the type of T2DM, an exercise protocol specifically designed to reverse muscle metabolic pathway alteration results in the optimal reduction of whole-body T2DM hallmarks, thus opening the way for precision exercises in T2DM patient care.

## Methods

### Animal care and ethics statement

All animal procedures were approved by the Institutional Animal Care and Use Committee of the University of Paris Cité (APAFIS#31064-20210310162620). Male (M) and female (F) mice with the following genetic backgrounds have been purchased from the Jackson Laboratory: 129S1/SvImJ (129/Sv), C57Bl/6J (C57), FVB-Tg Ckm-IGF1R*K1003R)1Dlr/J (MKR), and FVB/Nrj (FVB) used as control for MKR mice. The T2DM genetic group (MKR) and Control (CTRL) groups received autoclaved and irradiated normal chow diet (18% kCal fat, 24% protein, 58% carbohydrate; 3.1 kCal/g) while high-fat diet (HFD) and high-fat diet/streptozotocin (HFD/STZ) groups were fed with 60% kCal fat, 20% kCal from protein (5.24 kcal/g) for 14 or 30 weeks. No STZ group was constituted in this study since only the combination with HFD is known to induce T2DM (Yin et al. 2020). The high-fat diet/streptozotocin groups were also treated with STZ at week 4 as previously described (Gilbert et al. 2011; Wang et al. 2013; Masson et al. 2009). Mice were euthanized at 20 or at 36 weeks for tissue sampling as previously described (Carbone et al. 2012).

### Measurement of glucose homeostasis

Glycaemia was measured in a blood droplet obtained from the tail vein using a glucometer (Accu-Chek Performa, Roche), as previously described (Walker et al. 1990). For the glucose (IPGTT) and insulin (IPITT) tolerance tests, all mice were fasted for 5 h and the blood glucose levels were measured at T0, then 5, 20, 60, 90, and 120 min after the intraperitoneal injection of either glucose (SIGMA) at 2g/kg of body weight or insulin (Actrapid™, Novonordisk) at 0.75IU/Kg of body weight diluted in 0.9% NaCl. The IPGTT and IPITT were performed at 32 weeks of age only, before the sacrifice, and with 76 h of delay between both.

### Body assessment

Body composition was evaluated by Time Domain Nuclear Magnetic Resonance (TD-NMR) with a LF-90 instrument (Bruker Optics, Inc.). Fat to lean mass ratio (FLMR) was calculated to determine pre-obese (0.466) and obese (0.658) status through thresholds in association with body mass index (BMI), as previously described (Woo et al. 2013).

### Metabolomic analysis by nuclear magnetic resonance (NMR) spectroscopy

#### Sample preparation

50mg of muscles tissues were prepared for extraction of metabolites. All were grinding with 400 µL of ice-cold methanol and 65 µL of ice-cold water and two stainless steel beads. The samples were systematically vortexed between each solvent addition. A volume of 200 µL of ice-cold chloroform, then 200 µL of ice-cold water and finally 200 µL of ice-cold chloroform were further added. The samples were then left on ice for 15 min, and centrifuged at 2000 rpm for 15 min. The upper methanol/water phase (containing polar metabolites) and the lower chloroform phase (containing lipophilic compounds) were transferred into separate vials. Solvents were removed under a stream of nitrogen for 30 min. The water phases were put in the freeze dryer one night. The dry water phase samples were resuspended in 600 µL of NMR buffer (100 mM sodium phosphate buffer pH 7.4, in D2O, with 3.57 mM TSP and 6 mM NaN3) to obtain the polar phase extract. The lipophilic phases were resuspended in 600 µL of deuterated NMR solvent (2:1 mixture of pure CDCl3 and CDCl3 containing 0.03% of TMS, to obtain a final solution of 0.01% of TMS) and then vortexed. After centrifugation (5 min), 600 µL of the supernatant was directly transferred into an NMR tube to prepare the organic phase extract.

#### NMR measurements

Polar samples were measured at 300 K on a Bruker Avance II 500 MHz spectrometer (Bruker BioSpin, France) equipped with a SampleXpress automation sample changer and a cryogenic 5 mm 13C/1H dual probe with Z-gradient.

Lipophilic samples were recorded on a 600 MHz Bruker Avance IVDr spectrometer equipped with a dual 1H/13C probe head, and a SampleJet handler operating at room temperature. The operating software was TopSpin 4.1.1. The sample temperature during NMR analyses was set to 300 K.

The spectra were acquired using a classical 1D 1H experiment used for the metabolomics analysis of biofluids by NMR (Beckonert et al. 2007) (1D-NOESY pulse sequence with presaturation for water suppression).

The data processing was performed using TopSpin 4.0 (Bruker BioSpin, France). The spectra were phased, baseline corrected, and referenced to TSP. Additional processing and bucketing of the 1H-NMR spectra was performed using NMRProcFlow (Jacob et al. 2017). Each 1D spectrum was reduced using variable size bucketing. The different normalization methods proposed by NMRProcFlow were tested. The constant sum normalization of the data matrix was found to give the best performances in the data analysis.

#### Data analysis

Multivariate data analysis was performed with SIMCA 17.0 software (Umetrics AB, Umea Sweden). An autoscaling (UV) or Pareto scaling (PAR) was used prior to the analysis of the data. An overview of the results has been generated with unsupervised analysis using the PCA method to identify outliers and perform quality control on the dataset. Based on these results, group classifications were established from the samples to generate supervised analysis. O-PLS-DA models were calculated to identify discriminant metabolites.

### RT-qPCR analysis

Quadriceps mouse tissues were collected in liquid nitrogen and RNA was extracted using manufacturer protocol with TRizol reagent (Invitrogen, Life Technologies, Saint-Aubin, France). Dnase treated 1 μg of mouse RNAs was reverse-transcribed with oligodT using reverse transcriptase Improm II (Promega France, Charbonnières, France). Quantitative real time PCR was performed with standard protocols using SYRB Green ROX detector in ABIPrism 7000 (ABgene, Courtaboeuf, France). Specific primers were used at 100 nM (Table 1). The relative amounts of cDNA in each sample were determined on the basis of the threshold cycle (Ct) for each PCR product and normalized to RPL13 and 26s mRNA for all mouse samples. These housekeeping genes have been determined as best internal controls using Bestkeeper (Pfaffl et al. 2004) algorithms. The analysis was done relatively to control samples and given by 2^−ΔΔCt^. The fold change value of each individual is calculated as follows: the individual in the relevant control group with the intermediate 2^−ΔΔCt^ value compared with the other individuals in the control group is taken as a reference and set at 1. The fold change of the other individuals, whatever the group, is calculated in relation to this reference. The values obtained are used to calculate the mean per group and the standard deviation*.*

**Table 1:** Primers sequences

Acronym	Name	Forward 5′→3′	Reverse 5′→3′
26S	40S ribosomal protein S26	AGAAGAAACAACGGTCGCGCCAAA	GCGCAAGCAGGTCTGAATCGTG
RPL13	Ribosomal protein L13	AGGGGCAGGTTCTGGTATTG	TGTTGATGCCTTCACAGCGT
GLUT4	Glucose transporter 4	CTGCAAAGCGTAGGTACCAA	CCTCCCGCCCTTAGTTG
PFKM	6-phosphofructokinase, muscle type	AGATCGTAGACGCCATCACC	GGCCCATCACTTCTAACACAA
GAPDH	Glyceraldehyde 3-phosphate dehydrogenase	ATCTTCTTGTGCAGTGCCAG	TTTGCCACTGCAAATGGCAG
PDK2	Pyruvate dehydrogenase kinase isoform 2	TCTTTCACCACATCAGACACG	TGGACCGCTTCTACCTCAG
PDK4	Pyruvate dehydrogenase kinase isoform 4	ATGTGGTGAAGGTGTGAAG	TGTGATGTGGTAGCAGTAGTC
MCT1	Monocarboxylate transporter 1	GTGACCATTGTGGAATGCTGC	GTCTCCTTTGGCTTCTCGTCG
CD36	Cluster of differentiation 36	ATTAATGGCACAGACGCAGC	TTCAGATCCGAACACAGCGT
VLDLR	Very-low-density-lipoprotein receptor	GAGCCCCTGAAGGAATGCC	CCTATAACTAGGTCTTTGCAGATATGG
CPT1	Carnitine palmitoyltransferase 1	TCTTCTTCCGACAAACCCTGA	GAGACGGACACAGATAGCCC
ACC1	Acetyl-CoA carboxylase 1	GCCTCTTCCTGACAAACGAG	TGACTGCCGAAACATCTCTG
ACC2	Acetyl-CoA carboxylase 2	GAGCTGCTGTGTAAACACGAGATTGCT	CTGGTGCCGGCTGTCCTC
DGAT2	Diglyceride acyltransferase 2	AGTGGCAATGCTATCATCATCG	AAGGAATAAGTGGGAACCAGATCA
PGC-1α	Peroxisome proliferator-activated receptor gamma coactivator 1-alpha	AGACGGATTGCCCTCATTTGA	GGTCTTAACAATGGCAGGGTTT
UCP3	Mitochondrial uncoupling protein 3	ATGAGTTTTGCCTCCATTCG	GGCGTATCATGGCTTGAAAT
ATF4	Activating transcription factor 4	CCTGAACAGCGAAGTGTTGG	TGGAGAACCCATGAGGTTTCAA
SLC3A2	Solute carrier family 3 member 2 large neutral amino acid transporter	GAGTTCTTGGTTGCAGGACG	AATTTGCTGCAGGTCAGAGGA
SLC36A1	Proton-coupled amino acid transporter 1	AAAACCATGGCTAGGGGTCG	CGAAGCCTCTGTGTGGACAT
SLC7A5	Large neutral amino acids transporter small subunit 1	CTCATCATTCGGCCCTCCTC	TCGCAGCCTTTACGCTGTAG
MuRF1	E3 ubiquitin-protein ligase TRIM63	GCAGGAGTGCTCCAGTCG	TCTTCGTGTTCCTTGCACAT
Atg1	AuTophaGy related 1	AGTGAGGACCGGCTACTGTG	GATCAAACGCTTGCGAATCT

List of studied genes, including their acronyms, full names, and corresponding forward and reverse primer sequences, allowing the assessment of their expression through classical RT-qPCR

### Swimming-based exercise protocols

At 20 weeks of age, 12 HFD-induced male mice and 12 HFD-STZ-induced C57 mice (both male and female) were divided into three subgroups for a 12-week swimming-based training program using our rodent counter-flow swimming-pool, as previously described (Grondard et al. 2008; Deforges et al. 2009). Each subgroup, consisting of 4 animals, followed a specific exercise protocol conducted three times a week. The T2DM groups were further divided into untrained (UTG), high-intensity training (TP1—20 min at 80% max speed + 2 min at max speed), combined high and low-intensity training (TP2—15 min at 80% max speed, 40 min at 50% max speed, 2 min at max speed), and low-intensity training (TP3—60 min at 50% max speed) subgroups.

### Sensorimotor and performance assessment

The anaerobic performance of mice was evaluated biweekly through a maximum speed test, measuring the duration each animal sustained maximum water flow.

Aerobic power, assessed monthly, involved an incremental test with water flow increasing in 2-min steps (0 L min^−1^ to the animal's maximum) at 0, 1, 2, 3, 3.5, 4, 4.5, and 5 L min^−1^. The maximal time of each animal was recorded.

Coordination and sensorimotor behavior were assessed monthly using a beam walk test, as previously described (Hichor et al. 2017), with three trials per animal. Measurements included speed (m s^−1^) and the total number of mistakes (slips) without falling.

Nociceptive sensitivity was evaluated through a hot plate test. Mice were placed on a 55 °C heating plate for a maximum of 30 s., as previously described (Menéndez et al. 2002). The time until nociception behaviors (e.g., jumping, hind paw-licking) were recorded.

The motor nerve conduction velocity (MNCV) test, conducted before sacrifice, as previously described (Pollari et al. 2018), involved measuring compound muscle action potentials via supra-maximal stimulation. Electrodes were placed at the sciatic notch (stimulating), aligned with the quadriceps muscle (recording), near the Achilles tendon (reference), and on the mouse's side (ground). Latency and peak-to-peak amplitude were determined.

### Statistical analysis

Results from all experiments are expressed as mean ± standard deviation (SD). Following the instructions from ARRIVE2.0 guideline and the national ethical recommendation for comparisons of multiple groups and conditions, we determine the minimum number of animal per group needed to achieve a 70% of statistical power using Sealed Envelope Ltd. 2012, Power calculator for continuous outcome superiority trial (https://www.sealedenvelope.com/power/continuous-superiority/) with the random blood glucose as main outcome. Following recommendations, for multiple groups comparison statistical significance was assessed performing a non-parametric One-way ANOVA test, a Kruskal–Wallis test, followed by a post hoc Dunnett’s test. For two groups comparisons, statistical signifiance was performed by a non-parametric Mann–Whitney test. Statistical significance was determined as a probability (P value) of less than 0.05. All statistical analysis was performed with Prism 8 software (GraphPad Software).

## Results

### Diabetic induction in mice differentially alters body weight and body composition depending on genetic background and sex

**Fig. 1:**
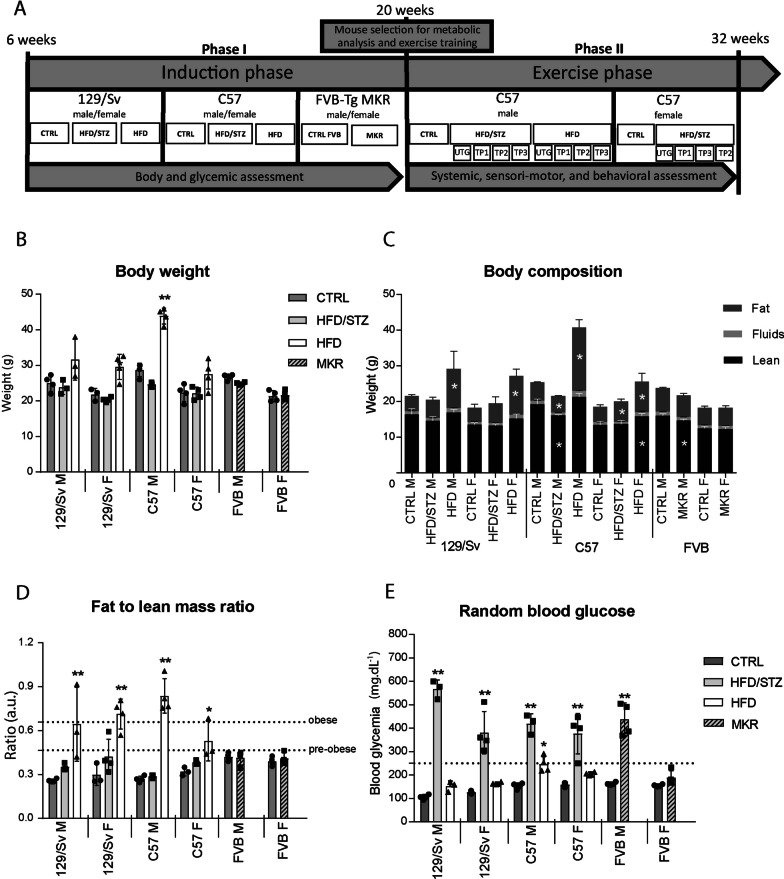
The background, sex, and method of T2DM induction significantly influence body weight, body composition, obesity status, and glycemia in mice. A schematic representation of the study design (**A**). Body weight assessment (**B**) was conducted in male (M) and female (F) mice induced with HFD/STZ (HFD/STZ) in both 129/Sv and C57 strains, as well as HFD-induced (HFD) mice in both 129/Sv and C57 strains, along with MKR mice from the FVB background, all evaluated at 20 weeks of age. Body composition, determined by NMR, included fat mass, total body fluids, and lean mass (**C**). The fat-to-lean mass ratio (**D**) was calculated using NMR data, with dotted lines indicating the thresholds for determining pre-obese (0.466) or obese (0.658) mice. Non-fasting blood glucose was measured using an Accu-check glucometer (**E**), and dotted lines indicate the hyperglycemia threshold (250 mg/dl). (n = 4 for all groups) All data are shown as mean ± standard deviation. Non-parametric One-Way ANOVA for all genetic backgrounds except MKR, non-parametric Mann–Whitney test; * indicate significance (with P < 0.05 *, < 0.005**) relative to controls (CTRL)

We first evaluated in phase 1 (Fig. 1A) the body weight in the different mouse models i.e. HFD/STZ- or HFD-induced 129/Sv and C57 mice, and MKR mice. We found that the treatment with HFD alone resulted in significant weight gain only in male C57 mice while the HFD/STZ combination did not induce weight gain, as previously shown (Yin et al. 2020) (Fig. 1B). As expected (Yin et al. 2020), the STZ treatment applied at 10 weeks of age induced a weight loss in both 129Sv and C57 genetic backgrounds, except for the female C57 (Yin et al. 2020), which is partly corrected until 20 weeks of age (Supplementary Fig. 1). Additionally, the MKR mice fed with normal chow did not display any weight modification when they where compared to FVB mice at 20 weeks of age (Fig. 1B).

Then, we investigated the mouse body composition (fat, fluid and lean) by NMR spectroscopy (Fig. 1C) and calculated the fat-to-lean mass ratio (FLMR) (Fig. 1D) to evaluate the obesity status of mice, according to Woo et al. (2013). While no change could be recorded in HFD/STZ-induced 129/Sv mice, with an FLMR under the pre-obese threshold (0.421 for males and females) (Fig. 1D), the impact of the HFD/STZ-induction in the C57 strain differed between sex, with an FLMR under the pre-obese threshold (mean ratio = 0.38) in females (Fig. 1D) despite a significant increase in fat mass (Fig. 1C), and an FLMR under the pre-obese threshold (mean ratio = 0.28) in males despite increased levels of fat, fluid and lean (Fig. 1C). In HFD-treated mice, we recorded a significant increase in fat mass (Fig. 1C), with an obese status reached in female 129/Sv and male C57 (mean ratios of 0.71 and 0.83, respectively), and a pre-obese status in male 129/Sv and female C57 (mean ratios of 0.64 and 0.52, respectively) (Fig. 1D). Finally, male and female MKR mice remained under pre-obese threshold (Fig. 1D), despite a significant decrease in fat, fluid and lean levels only in males (Fig. 1C).

Thus, taken together, these results suggested that T2DM-induced mice likely develop either obesity (HFD-induced female 129SV/1 and male C57BL/6 mice), a pre-obese status (HFD-induced male 129SV/1 and female C57BL/6 mice) or no obesity (male and female HFD/STZ-induced and MKR mice), thus giving the opportunity to compare the muscle metabolism in these different situations.

### Non-fasting blood glucose is differentially increased in pre-diabetic and diabetic mouse models.

We compared blood glucose levels in different mouse populations following HFD/STZ treatment. All mouse populations exhibited significant hyperglycemia indicative of T2DM (blood glucose levels exceeding 250 mg/dl), though with varying severity (Fig. 1E). Notably, in the 129/Sv strain, males induced by HFD/STZ displayed a 33% higher increase in blood glucose levels compared to females (566 mg/dl vs. 381 mg/dl, respectively). For HFD-induced, only C57 males reached a significant glycemic value indicative of T2DM (246 mg/dl) (Fig. 1E). In the MKR strain, only males became hyperglycemic (437 mg/dl in males, 188 mg/dl in females).

These results show that the different mouse populations differentially reached a T2DM compatible hyperglycaemia.

### The skeletal muscle metabolism is differentially altered in the diabetic mouse models

**Fig. 2:**
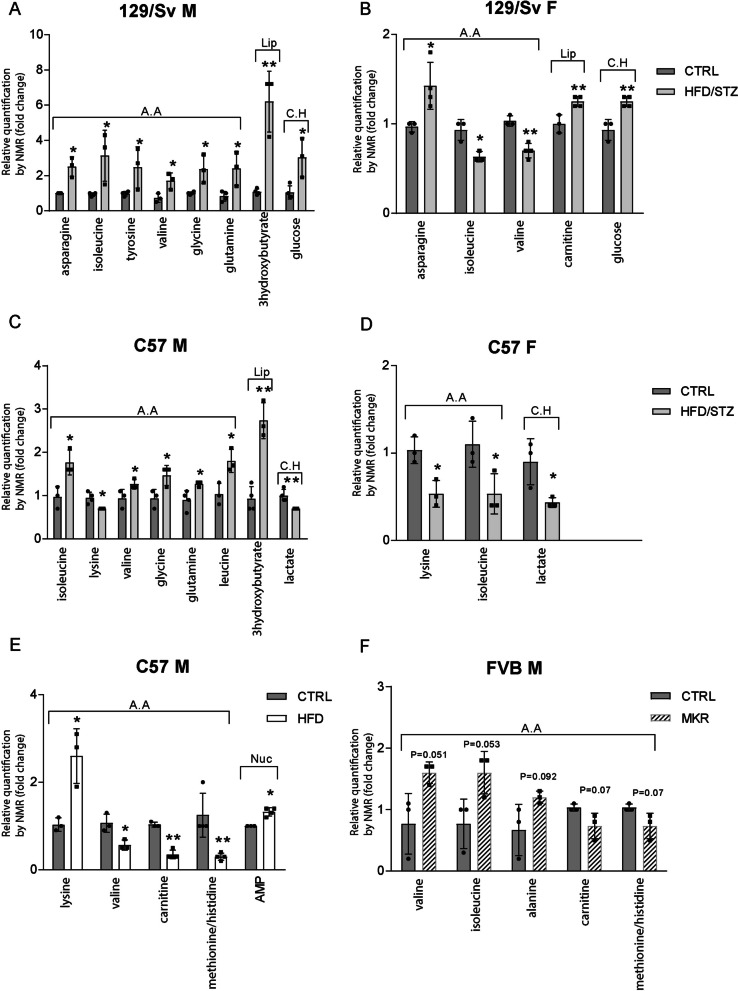
The skeletal muscle metabolism is differentially altered among various diabetic mouse models, influenced by genetic background, sex, and the mode of T2DM induction. Quadriceps muscle metabolomics at 20 weeks, evaluated by NMR, is presented as fold change compared to control mice. Results are shown for male (M) and female (F) HFD/STZ-induced (HFD/STZ) 129/Sv mice (**A**, **B**) and C57 mice (**C**, **D**), as well as male HFD-induced (HFD) C57 mice (**E**) and male MKR mice (**F**). Metabolite categories include AA (amino acids), Lip (lipid pathways), C.H (carbohydrate pathways), and Nuc (nucleotides). (n = 4 for all groups) All data are shown as mean ± standard deviation. Non-parametric Mann–Whitney test for all genetic backgrounds; * indicate significance (with P < 0.05 *, < 0.005**) relative to controls (CTRL)

We compared the muscle energy metabolism of relevant mouse populations exhibiting T2DM-compatible hyperglycemia associated with different obesity statuses. Using unbiased metabolomics based on nuclear magnetic resonance spectroscopy (NMR) we compared the concentration of main metabolites from the different energetic pathways of the quadriceps muscle of male (Supplementary Fig. 2A and 2C) and female (Supplementary Fig. 2B and 2D) HFD/STZ-induced 129/Sv (Supplementary Fig. 2A and 2B) and C57 (Supplementary Fig. 2C and 2D), male HFD-induced C57 (Supplementary Fig. 2E), and male MKR mice (Supplementary Fig. 2F), and we expressed the differences in fold change compared to controls (Fig. 2).

In HFD/STZ-induced 129/Sv mice, we observed muscular glucose accumulation (Fig. 2A and B) and a decrease in lactate concentrations in HFD/STZ-induced C57 mice (Fig. 2C and D), suggesting a depression in carbohydrate anaerobic catabolism. Additionally, in both strains, we noted a significant increase in 3-hydroxybutyrate, indicating an increased reliance on lipid catabolism for energy needs, particularly in males (Fig. 2A–C).

Interestingly, a sex-dependent alteration in amino acid (AA) homeostasis was found in mouse quadriceps, affecting both essential (EAA) and non-essential (NAA) amino acids (Fig. 2A–F). HFD/STZ-induced 129/Sv males displayed a significant increase in EAA and NAA levels, while females showed low concentrations of EAA and high concentrations of NAA (Fig. 2A, B). In HFD/STZ-induced C57 males, NAA increased, with contrasted alterations in EAA levels, while females displayed decreased EAA levels (Fig. 2C, D). Male HFD-induced C57 mice showed an overall decrease in EAA and NAA concentrations and an increase in lysine (Fig. 2E). In MKR males, AA metabolism appeared less disturbed, with only a tendency to increase EAA and NAA concentrations (Fig. 2F).

Taken together these results strongly suggest that, in the different diabetic mouse models, all the main metabolic pathways, including carbohydrates, lipids, and proteins are perturbed in the fast-twitch muscle *i.e.* the quadriceps, but with genetic background, gender, and type of induction specificities.

### The expression pattern of genes involved in energy metabolism is differentially altered in the muscle of diabetic mouse models, depending on the sex and the type of induction

**Fig. 3:**
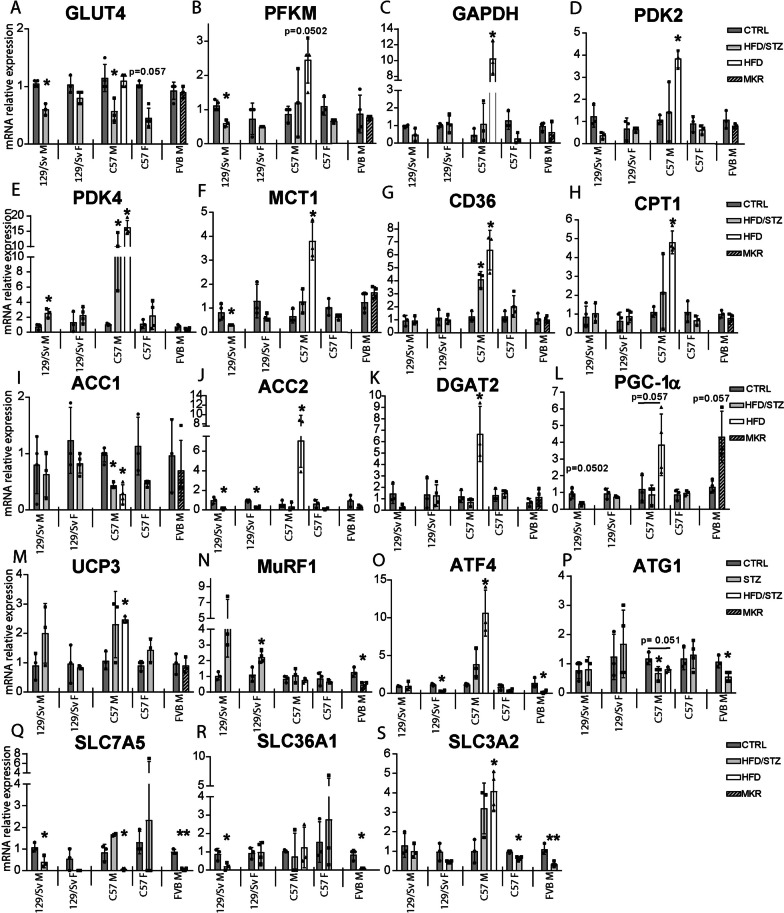
The mRNA expression levels of key components in muscle metabolic pathways exhibit selective alterations in distinct T2DM mouse models. Quantification of mRNA gene expression in (i) the carbohydrate pathway, including GLUT4 (**A**), PFKM (**B**), GAPDH (**C**), PDK2 (**D**), PDK4 (**E**), and MCT1 (**F**); (ii) the lipid pathway, including CD36 (**G**), CPT1 (**H**), ACC1 (**I**), ACC2 (**J**), and DGAT2 (**K**); (iii) mitochondrial metabolism, including PGC1α (**L**), and UCP3 (**M**); and (iv) amino-acid metabolism, including MuRF1 (**N**), ATF4 (**O**), ATG1 (**P**), SLC7A5 (**Q**), SLC36A1 (**R**), and SLC3A2 (**S**), was performed by RT-qPCR. Samples were obtained from male (M) and female (F) HFD/STZ-induced (HFD/STZ) 129/Sv and C57 mice, HFD-induced (HFD) male C57 mice, and male MKR mice from the FVB background at 20 weeks of age. mRNA expression levels were normalized to RPL13 and 26S mRNA. (n = 4 for all groups) All data are shown as mean ± standard deviation. Non-parametric Mann–Whitney test for all genetic backgrounds except C57 males, non-parametric One-Way ANOVA; * indicate significance (with P < 0.05 *, < 0.005**) relative to controls (CTRL)

In order to further investigate diabetes-induced alterations in mouse muscle metabolism, we evaluated by RT-qPCR the mRNA expression patterns of the main components of the carbohydrate, lipid, and amino-acid (AA) metabolic pathways. In terms of carbohydrate metabolism, male HFD/STZ-induced mice exhibited diminished GLUT4 expression alongside an increase in PDK4 (Fig. 3A and E). Notably, male 129/Sv mice displayed reduced levels of PFKM and MTC1 (Fig. 3B and F). In female HFD/STZ-induced mice, especially in C57, there was a decline tendency in GLUT4 (Fig. 3A). Conversely, HFD-induced male C57 mice demonstrated heightened expression of PFKM, GAPDH, PDK2, PDK4, and MCT1, suggesting a potential hindrance in pyruvate transfer to mitochondria (Fig. 3B–F). Thus, while the muscle carbohydrate catabolism was severely depressed in HFD/STZ-induced diabetic males, it was quite normal in females and MKR, and likely enhanced in HFD-induced C57BL/6 male mice.

Turning to lipid metabolism, male HFD/STZ-induced mice displayed an overall increase in lipid uptake, with elevated CD36 in C57, coupled with reduced fatty acid biosynthesis (ACC2 down-regulation in 129/Sv and ACC1 in C57) (Fig. 3G, I and J). Female HFD/STZ-induced mice solely exhibited a decrease in fatty acid biosynthesis (Fig. 3I and J). In HFD-induced C57 males, there was significant up-regulation of CD36, CPT1, ACC2, and DGAT2, alongside ACC1 down-expression (Fig. 3 G, H, J, K and I) I. These data suggest that the muscle lipid metabolism globally adapt in two different ways either coherent with a boost in β-oxidation and inhibition in fatty acid storage, for non-obese mice or a boost in lipid biosynthesis in diabetic obese mice.

Regarding amino acid metabolism, there was a widespread alteration in AA transport gene expression. Reduced expression was observed for SLC7A5 in HFD/STZ-induced male 129/Sv, HFD-induced male C57, and male MKR mice (Fig. 3Q). SLC36A1 demonstrated decreased expression in HFD/STZ-induced male 129/Sv and MKR mice (Fig. 3R). SLC3A2 exhibited decreased expression in HFD/STZ-induced female C57 and male MKR mice, but increased expression in HFD/STZ-induced male C57 and HFD-induced male C57 (Fig. 3S). ATF4 expression increased in HFD/STZ- and HFD-induced male C57 mice, accompanied by a decrease in ATG1 expression (Fig. 3O and P). HFD/STZ-induced female 129/Sv showed increased MuRF1 expression and decreased ATF4 expression (Fig. 3N and O). In MKR muscles, ATF4 and ATG1 expression decreased, indicating a disruption in the balance between protein synthesis and degradation (Fig. 3O and P). These results provide the first lines of evidence that the traffic of free AA, as well as, their use to synthesize proteins, are compromised in diabetic muscles.

Taken together, these data suggested a differential and global adaptation of muscle metabolism to the different diabetic conditions, strongly depending on the gender and the type of induction. This led us to draw three different signatures of muscle metabolism adaptation to T2DM, *i.e.* non-obese HFD/STZ-induced male and female C57 mice, characterized by a β-oxidation shift with depressed glycolysis, and either (1) increased (males) or (2) decreased (females) free amino acid concentrations, and (3) obese HFD-induced male C57 mice, representing carbohydrate, lipid, and protein hypermetabolism.

### Systemic T2DM hallmarks are differentially improved by specific exercise programs

We examined in phase 2 (Fig. 1A) the effects on T2DM metabolic hallmarks of three different exercise paradigms (TP1, 2, and 3, detailed in methods) aimed at specifically targeting the metabolic impairments. To do this, we selected the three mouse populations showing a specific metabolic signature, which in addition shared the same genetic background i.e. C57, to avoid bias.

**Fig. 4:**
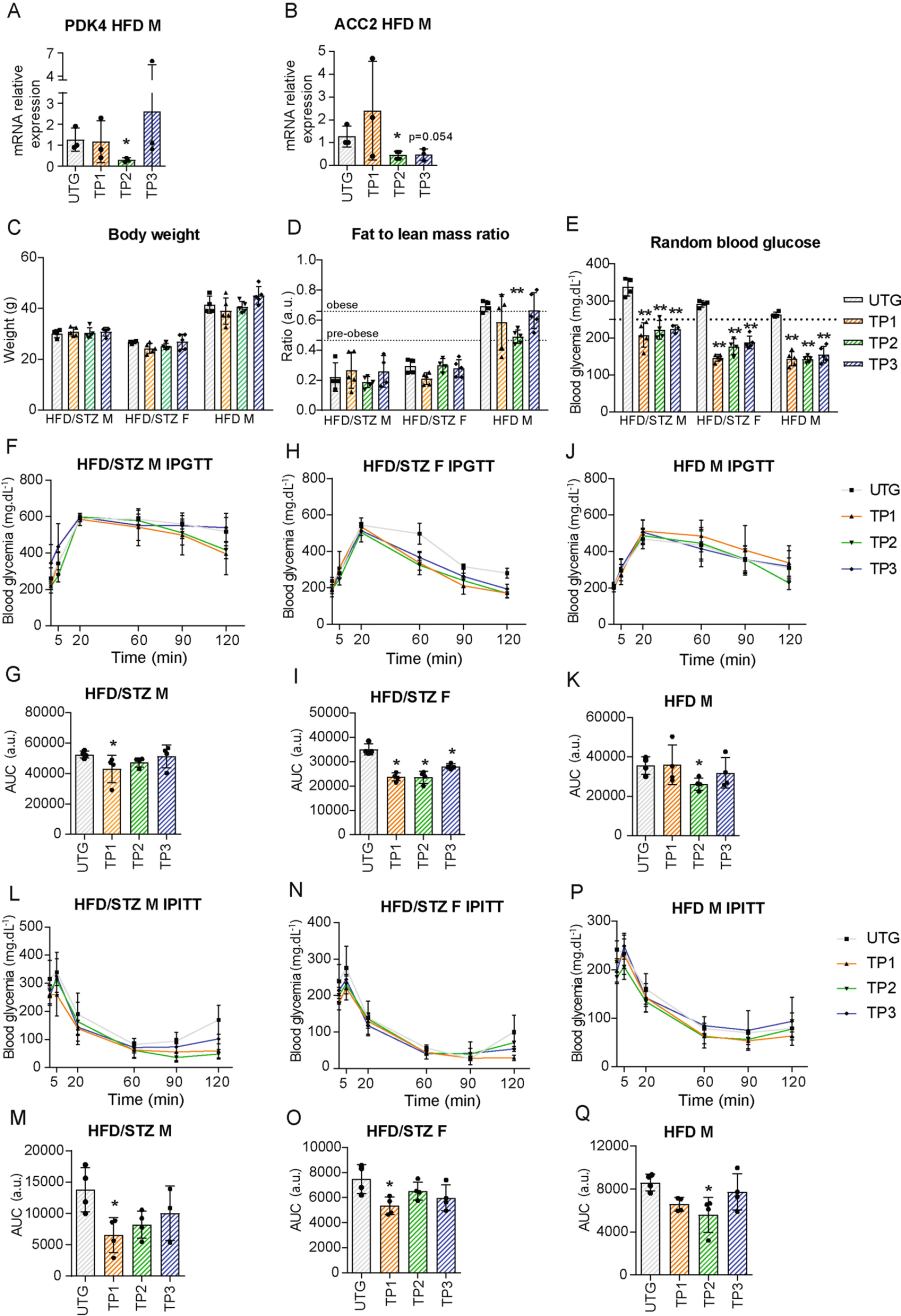
Differential effects of specific exercise programs on body assessment and systemic diabetes hallmarks in various T2DM mouse models. High-intensity TP1, a combination of high and low intensity TP2, and low-intensity TP3 swimming protocols were evaluated in HFD/STZ-induced male (HFD/STZ M) and female (HFD/STZ F) C57, and HFD-induced male (HFD M) C57 mice at 32 weeks of age. Results were compared to relevant untrained diabetic groups (UTG). Quadriceps mRNA gene expression levels of PDK4 (**A**) and ACC2 (**B**) in HFD-induced muscles were quantified by RT-qPCR and normalized with RPL13 and 26S mRNA. Body weight (**C**) was directly recorded using a scale. The fat-to-lean mass ratio (**D**) was calculated from NMR Minispec LF90 data, with dotted lines indicating thresholds for pre-obese (0.466) or obese (0.658) mice. Random blood glucose (**E**) was measured using the Accu-check glucometer, with dotted lines indicating the hyperglycemia threshold (250 mg/dl). Glucose tolerance (IPGTT) (**F**, **H**, **J**) and insulin tolerance (IPITT) (**L**, **N**, **P**) tests were conducted, and statistical analysis of the area under the curve (AUC) was determined for IPGTT (**G**, **I**, **K**) and IPITT (**M**, **O**, **Q**). (n = 5 for all groups) All data are shown as mean ± standard deviation. Non-parametric One-Way ANOVA for all groups; * indicate significance (with P < 0.05 *, < 0.005**) relative to untrained (UTG)

We first assessed the effectiveness of each exercise program in targeting altered metabolic pathways, using HFD-induced C57 male muscles as a proof-of-concept. As expected, TP2 significantly reduced PDK4 (Fig. 4A) and ACC2 (Fig. 4B) expression, TP3 reduced ACC2, and TP1 had no effect.

We then explored the impact of each exercise on mouse obesity status. If no change in body weight was observed (Fig. 4C; Supplementary Fig. 3A), a significant improvement in FLMR of obese mice subjected to TP1 and TP2 was noticed, reaching a pre-obese or non-obese threshold (mean ratio 0.58 for TP1 and 0.49 for TP2), with no effect for TP3 (mean ratio 0.66) (Fig. 4D; Supplementary Fig. 3B). No changes in body weight or FMLR were observed in non-obese mouse models (Fig. 4C and D; Supplementary Fig. 3A and 2B).

As anticipated, all exercise paradigms significantly reduced non-fasting glycaemia, reaching values below the hyperglycemia threshold (250 mg/dl) in all mouse models (Fig. 4E; Supplementary Fig. 3C). Glucose sensitivity was improved by TP1 and TP2 in non-obese STZ/HFD-induced mice, with optimal effects from TP1 (Fig. 4F–I; Supplementary Fig. 3D–G), and by TP2 and TP3, with TP2 providing optimal effects in obese HFD-induced mice (Fig. 4J and K; Supplementary Fig. 3D and 3E). TP1 efficiently restored insulin sensitivity in non-obese mouse models (Fig. 4L–O; Supplementary Fig. 3H–K), whereas TP2 significantly improved insulin sensitivity only in obese males (Fig. 4P and Q; Supplementary Fig. 3H and 3I).

Thus, all these data show, for the first time, that specific exercise can target specific T2DM-induced metabolic impairment, resulting in optimal benefits for T2DM mouse models.

### The sensorimotor performances of the different T2DM mouse models are differentially improved by the specific exercise programs

**Fig. 5:**
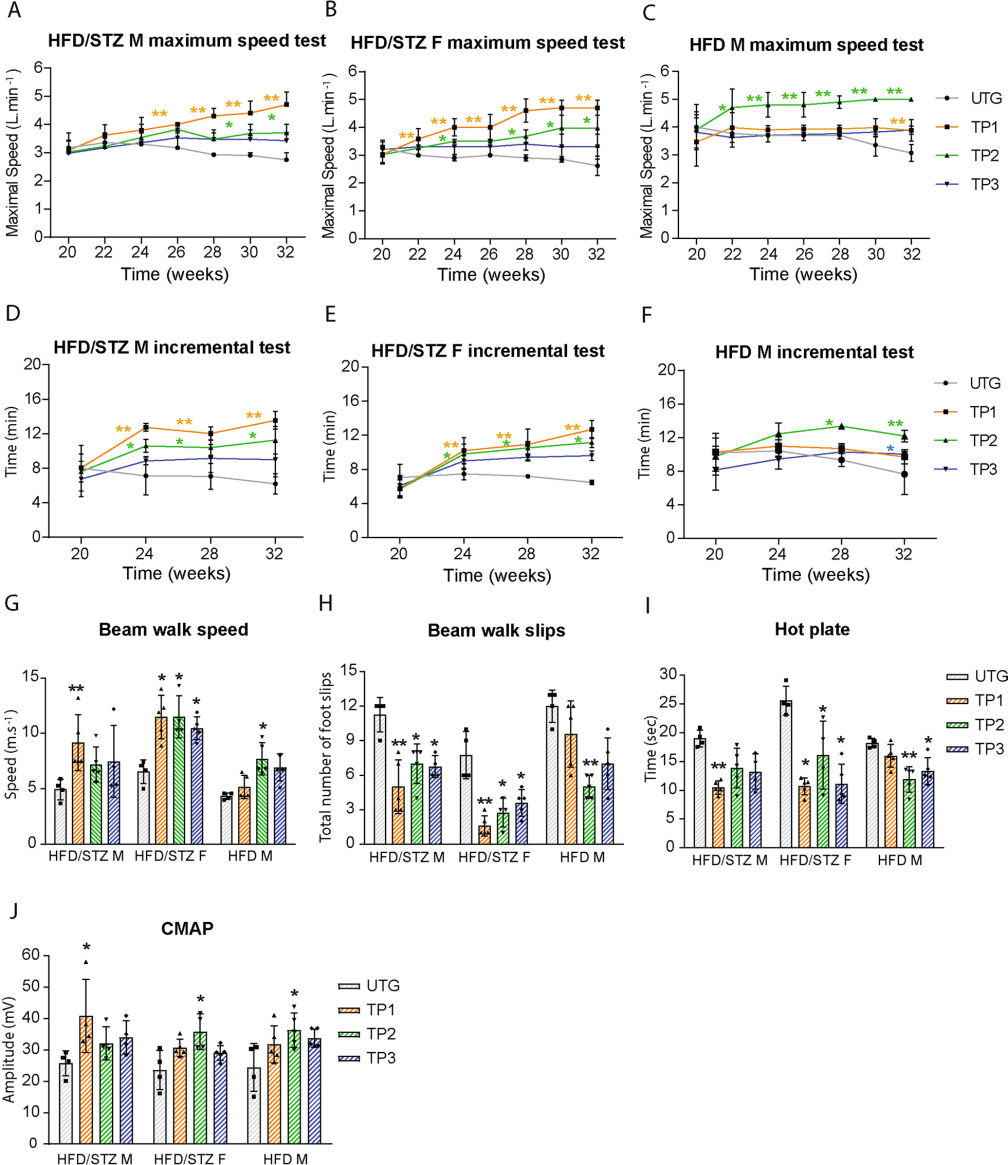
Precision exercise programs are optimal in limiting T2DM hallmarks in the different mouse models. The maximum speed test (**A**–**C**) and incremental test (**D**–**F**) were assessed from the age of 20 weeks to the age of 32 weeks in HFD/STZ induced male (HFD/STZ M) and female (HFD/STZ F) C57 mice and HFD-induced male (HFD M) C57 mice, submitted to personalized exercises (TP1, TP2 and TP3), and compared to untrained group (UTG). The motor coordination was assessed by (i) the beam walk test, during which the speed (**G**) and foot slips (**H**) were measured, (ii) the thermal sensitivity hot plate test (**I**) and (iii) CMAP amplitude (**J**) in all trained groups at 32 weeks of age, compared to untrained (UTG). (n = 5 for all groups) All data are shown as mean ± standard deviation. Non-parametric One-Way ANOVA for all groups; * indicate significance (with P < 0.05 *, < 0.005**) relative to untrained (UTG)

We finally examined the effects of the different exercise paradigms on T2DM mouse sensorimotor behaviour. Using the maximum speed test to assess the mouse anaerobic performances, we found that, for non-obese T2DM mouse models, only the TP1 program was able to increase the maximum speed from week 20 (mean of 3 L min^−1^ in males, 3.2 L min^−1^ in females) to week 32 (similar mean of 4.7 L min^−1^) (Fig. 5A and B). By contrast, in obese diabetic mice, despite the global positive effects of all exercise programs, it was TP2 that provided the optimal effects on anaerobic performances, with an increase in maximum speed from week 20 (mean 3.8 L min^−1^) to week 32 (mean 5 L min^−1^) (Fig. 5C).

Then, using the incremental test, we found that TP1 was optimal in non-obese T2DM mouse models, as it was able to increase the fitness power from week 20 (mean 8 min 26 s in males, and 5 min 17 s in females) to week 32 (mean of 13 min 32 s in males, and 12 min 41 s in females) (Fig. 5D and E) compared to untrained counterparts (mean of 6 min 17 s in males and 6 min 28 s in females). By contrast, TP2 was found optimal in increasing fitness power in obese T2DM mice from week 20 (mean 9 min 45 s) to week 32 (mean 12 min 6 s) (Fig. 5F).

We next investigated the mouse motor coordination, using the beam walk test, and found that TP1 was optimal in improving walking coordination in HFD/STZ-induced male and female C57 mice, notably by limiting the walking speed decline and the number of foot slips (Fig. 5G and H; Supplementary Fig. 3L, and 3M). As expected, TP2 provided the optimal benefits in HFD-induced male C57 mice, with improvement in the walking speed and in the foot slips (Fig. 5G and H).

Finally, using thermal nociception and motor-nerve conduction velocity tests, we found that while T2DM induction proved to significantly decrease thermal sensitivity and motor nerve conduction amplitude in all untrained mouse groups compared to controls (Supplementary Fig. 3N and 3O), exercise differentially limited the sensorimotor alterations with, according to the motor performances, an optimal effect for TP1 in HFD/STZ-induced male and female C57 mice and for TP2 in HFD-induced male C57 mice (Fig. 5I and J).

Taken together, these behavioral data provide the first lines of evidence that the specific exercise-induced improvement of metabolic parameters in T2DM mice is associated with significant benefits in terms of sensorimotor behavior.

## Discussion

In the present paper, we report that designing physical exercise protocols aimed at improving the specific muscle metabolic alterations resulted in the optimal reduction of systemic T2DM hallmarks, thus opening the way for the use of precision exercises in T2DM patient care.

Based on a combination of metabolomics by NMR and RT-qPCR in the fast-twitch quadriceps muscle in obese or non-obese diabetic mice, we provide here a set of different metabolic signatures, which include (i) a shift from glycolytic to β-oxidation, despite the fast-twitch phenotype of the quadriceps, in non-obese HFD/STZ-induced C57 mice, and (ii) an anaerobic carbohydrate hypermetabolism (overexpression of glycolysis enzymes and of PDK4) associated with an increase in the biosynthesis of lipids (overexpression of lipid transporters and lipid synthesis enzymes) and proteins (increase in the expression of AA transporters and of ATF4, decrease in free AA intramuscular concentration) in obese HFD-induced C57mice. These anabolic effects are likely associated with a boost in mitochondrial biogenesis (over-expression of PGC1α). Between these two extreme situations, the metabolic signatures induced under the other conditions (HFD/STZ-induced female mice and MKR mice), globally constituted intermediate profiles. Indeed, a signature of glucose resistance was found in muscles from non-obese mice regardless of the strain (129/Sv or C57) and sex. However, contrarily to the male HFD/STZ-induced mice, there is no clear evidence of a shift to β-oxidation neither significant change in lipolysis enzyme expression pattern nor increase in 3hydroxybutyrate levels. Noteworthy, the muscles from female HFD/STZ-induced 129/Sv mice displayed significant signs of depressed protein synthesis (decrease in ATF4 expression, global increase in free AA concentration) and atrophy (increase in MuRF1 expression). Finally, the quadriceps of male MKR mice show a metabolic signature mainly characterized by the deregulation of the pathways regulating protein synthesis (decrease in AA transporters, ATF4 and ATG1 expression levels, and tendency for intra-muscular increase in free AA concentrations).

One intriguing finding of the present study is the global disruption of AA homeostasis in the diabetic muscles, with variations depending on the mouse models. Decreased glycolysis and increased β-oxidation were previously shown to increase Branched-Chain AA (BCAA: valine, leucine, isoleucine) concentrations in diabetic muscles (Holeček 2020), concurring to our results in the quadriceps of HFD/STZ-induced male mice. Besides BCAA, we report here that the concentration of essential AA (EAA: isoleucine and valine in both strains and leucine specifically in the C57 strain) as well as non-essential AA (NEAA: glutamine, glycine in both strains, and asparagine, tyrosine specifically in the 129/Sv strain) increased in quadriceps muscles. This global increase in intramuscular free AA concentrations is unlikely due to depressed protein synthesis (AFT4 over-expression in C57 males), activation of autophagy (ATG1 down-regulation in C57 and MKR males), or excessive AA uptake (slight modifications in transporter expression profiles). Intriguingly, the alteration of AA homeostasis in diabetic muscles is dependent of sex. Indeed, the BCAA concentrations decreased in the quadriceps of HFD/STZ-induced female mice, as did the EAA (isoleucine in both strains, valine in 129/Sv, and lysine in C57). These decreases are associated with molecular signs of protein synthesis inhibition (downregulation of ATF4) and muscle atrophy (overexpression of MuRF1) in the 129/Sv strain, and a deregulation of AA transporters in the C57 strain. Besides, the global decrease in AA intramuscular concentrations in females, contrarily to the males, may result from the use of AA as substrates to sustain energy needs, in a context of glucose resistance without compensation by β-oxidation. Although the sex-dependent use of AAs as fuel has been already described in the liver of diabetic mice (Della Torre et al. 2018), to the best of our knowledge, a sex-dependent metabolic adaptation in diabetic muscle was unexpected. In the obese HDF-induced male C57 mice, we found a global decrease in AA concentrations, supporting the hypothesis of an increased protein synthesis (significant ATF4 and SLC3A2 overexpression and tendency to ATG1 downregulation). Finally, a trend toward increased AA concentrations was recorded in MKR muscles, associated with defects in AA uptake (decreased SLC3A2 and SLC36A1 expression levels), protein synthesis (downregulation of ATF4) and autophagy (downregulation of ATG1).

Most importantly, we provide here the first lines of evidence that designing a training program on exercises aimed at targeting specific T2DM-altered metabolism pathways significantly optimizes the benefits of training on systemic diabetes hallmarks. Thus, while the pure high intensity protocol TP1 proved to be optimal for non-obese diabetic mice characterized by a metabolic shift to β-oxidation, the high/low intensity protocol TP2 was found optimal in obese diabetic mice, characterized by muscle hypermetabolism. Very interestingly, these specific improvements at the muscle metabolism were associated with optimal improvements of the main T2DM hallmarks at the systemic level, with significant increases in glucose and insulin sensitivities and sensorimotor function. In addition, our data provide molecular cues accounting for the benefits of including resistance to aerobic training to limit the risk of diabetes in human subjects with dyslipidemia (Ross et al. 2021). Noteworthy, we found that the glycaemia was indiscriminately decreased by all training paradigms, including the pure low intensity training TP3, suggesting the promotion of adaptive mechanisms independent of muscle response to exercise, such as the improvement of the pancreatic β-cells as previously proposed (Malin 2018). Obviously, future investigations are required to precisely investigate the mechanism underlying the impact of each exercise on the muscular energy metabolism specifically altered in the different T2DM mouse populations.

## Conclusion

Taken together, the present data indicate that diabetes-induced impairments of muscle metabolism depend on the type of diabetes, and that therapies aimed at specifically improving the affected metabolic pathways can result in significant positive effects on the major hallmarks of the disease at the systemic level. The question of transferring these findings to humans opens up the challenge of analysing muscle metabolism in patients by non-invasive means. One possible route could be spectral NMR targeting metabolic intermediates of major catabolic pathways, in order to identify the ones to target with precision exercises.

### Supplementary Information

Supplementary Material 1. **Supplementary Fig. 1.** The influence of the type of induction on body weight progression. The evaluation of body weight at 6, 10, 11, and 20 weeks of age of male (M) and female (F) HFD/STZ-induced (HFD/STZ) and HFD-induced 129/Sv, and C57 mice. The HFD/STZ groups were injected with multiple low dose of STZ at 10 weeks of age.

Supplementary Material 2. **Supplementary Fig. 2.** The metabolomic analysis of quadriceps muscle in the different T2DM mouse models indicated specific metabolic alterations. The evaluation of quadriceps muscle metabolomics at 20 weeks of age by nuclear magnetic resonance spectroscopy (NMR) in male (M) and female (F) HFD/STZ-induced (HFD/STZ) 129/Sv (A, B) and C57 (C, D) mice, HFD-induced (HFD) C57 (E) mice, and MKR male mice from FVB background (F). The diagrams represent the normalized values of diabetic groups in green (HFD/STZ, HFD, MKR) and control groups in red (CTRL), in relation to the centered mean spectral value at 0. The spectral figures represent the relative intensity (y axis) compared to the frequency of resonance of the NMR signal (x axis) of diabetic groups in green (HFD/STZ, HFD, MKR) compared to control groups in red (CTRL). (n = 4 for all groups) All data are shown as mean ± standard deviation. Non-parametric Mann–Whitney test for all genetic backgrounds; * indicate significance (with P < 0.05 *, < 0.005**) relative to controls (CTRL).

Supplementary Material 3.** Supplementary Fig. 3.** The sex and method of T2DM induction significantly influence body weight, obesity status, glycemia and sensorimotor function in mice. Body weight assessment (A) was conducted on the untrained groups of male (M) and female (F) mice induced with HFD/STZ (UTG HFD/STZ), as well as HFD-induced (UTG HFD) mice in comparison to CTRL groups. The fat-to-lean mass ratio (B) was calculated using NMR data, with dotted lines indicating the thresholds for determining pre-obese (0.466) or obese (0.658) mice. Non-fasting blood glucose was measured using an Accu-check glucometer (C), and dotted lines indicate the hyperglycemia threshold (250 mg/dl). Glucose tolerance (IPGTT) (D, F) and insulin tolerance (IPITT) (H, J) tests were conducted, and statistical analysis of the area under the curve (AUC) was determined for IPGTT (E, G) and IPITT (I, K). The motor coordination was assessed by i) the beam walk test, during which the speed (L) and foot slips (M) were measured, ii) the thermal sensitivity hot plate test (N) and iii) CMAP amplitude (O) in all untrained groups at 32 weeks of age, compared to controls (CTRL). (n = 5 for all groups). All data are shown as mean ± standard deviation. Non-parametric One-Way ANOVA for all C57 males, and non-parametric Mann–Whitney test for all C57 females; * indicate significance (with P < 0.05 *, < 0.005**) relative to controls (CTRL).

## Data Availability

All data needed to evaluate the conclusions in the paper are present in the paper and/or the additional files. Additional data related to this paper may be requested from the authors.

## Abbreviations

STZ: Streptozotocin
IPGTT: Intraperitoneal glucose tolerance test
IPITT: Intraperitoneal insulin tolerance test
UTG: Untrained group
TP1: Training protocol 1
TP2: Training protocol 2
TP3: Training protocol 3
MNCV: Motor nerve conduction velocity
FLMR: Fat-to-lean mass ratio
AA: Amino acid
EAA: Essential amino acid
NAA: Non-essential amino acid
