# Supplementary figures and images for: The analysis of the skeletal muscle metabolism is crucial for designing optimal exercise paradigms in type 2 diabetes mellitus
Source: Mol Med. 2024 Jun 10;30:80. doi: 10.1186/s10020-024-00850-7 (PMC11165837; doi:10.1186/s10020-024-00850-7)

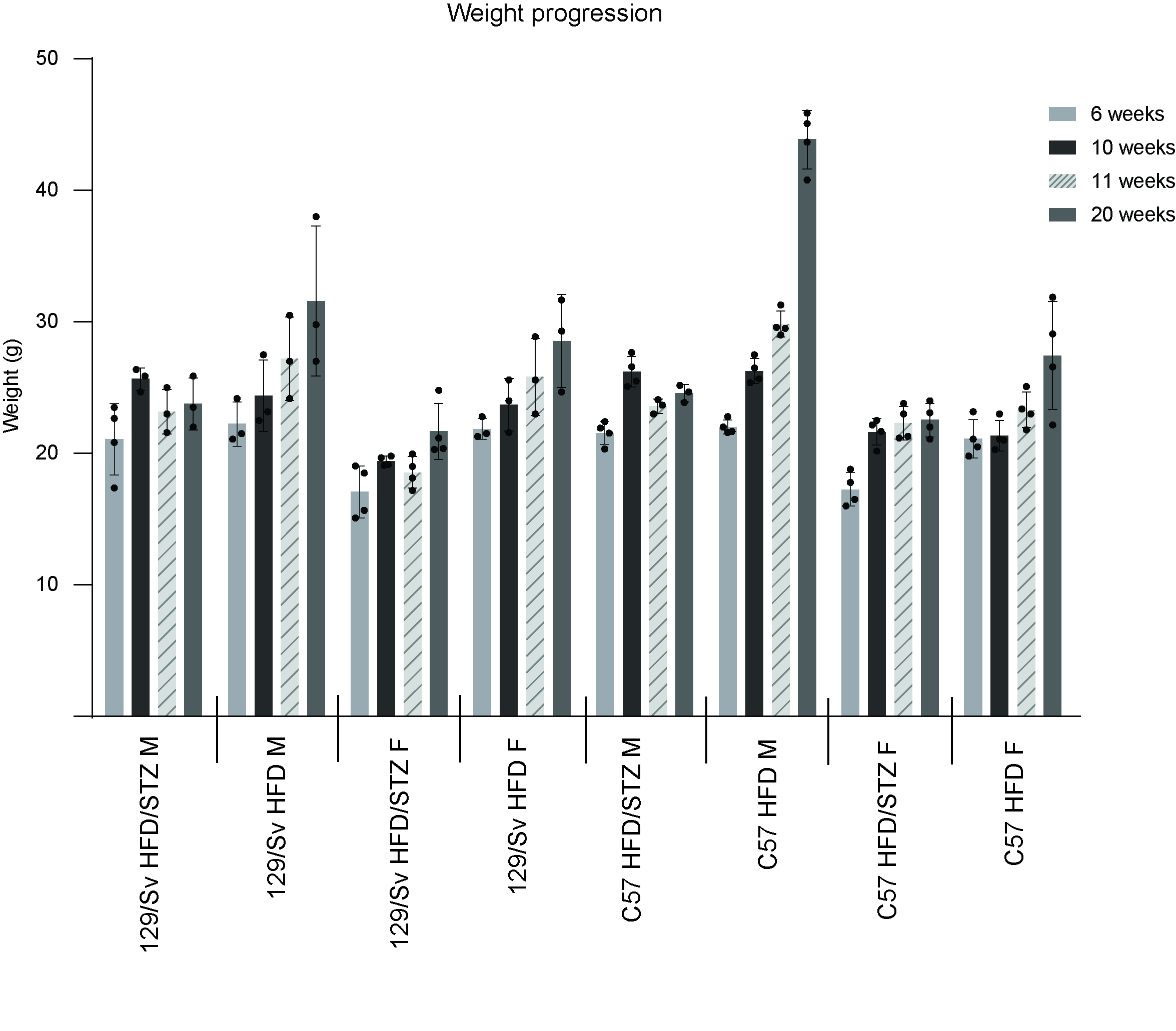

Supplement: Supplementary file 1 — Supplementary Material 1. Supplementary Fig. 1. The influence of the type of induction on body weight progression. The evaluation of body weight at 6, 10, 11, and 20 weeks of age of male (M) and female (F) HFD/STZ-induced (HFD/STZ) and HFD-induced 129/Sv, and C57 mice. The HFD/STZ groups were injected with multiple low dose of STZ at 10 weeks of age. [file 10020_2024_850_MOESM1_ESM.jpg]

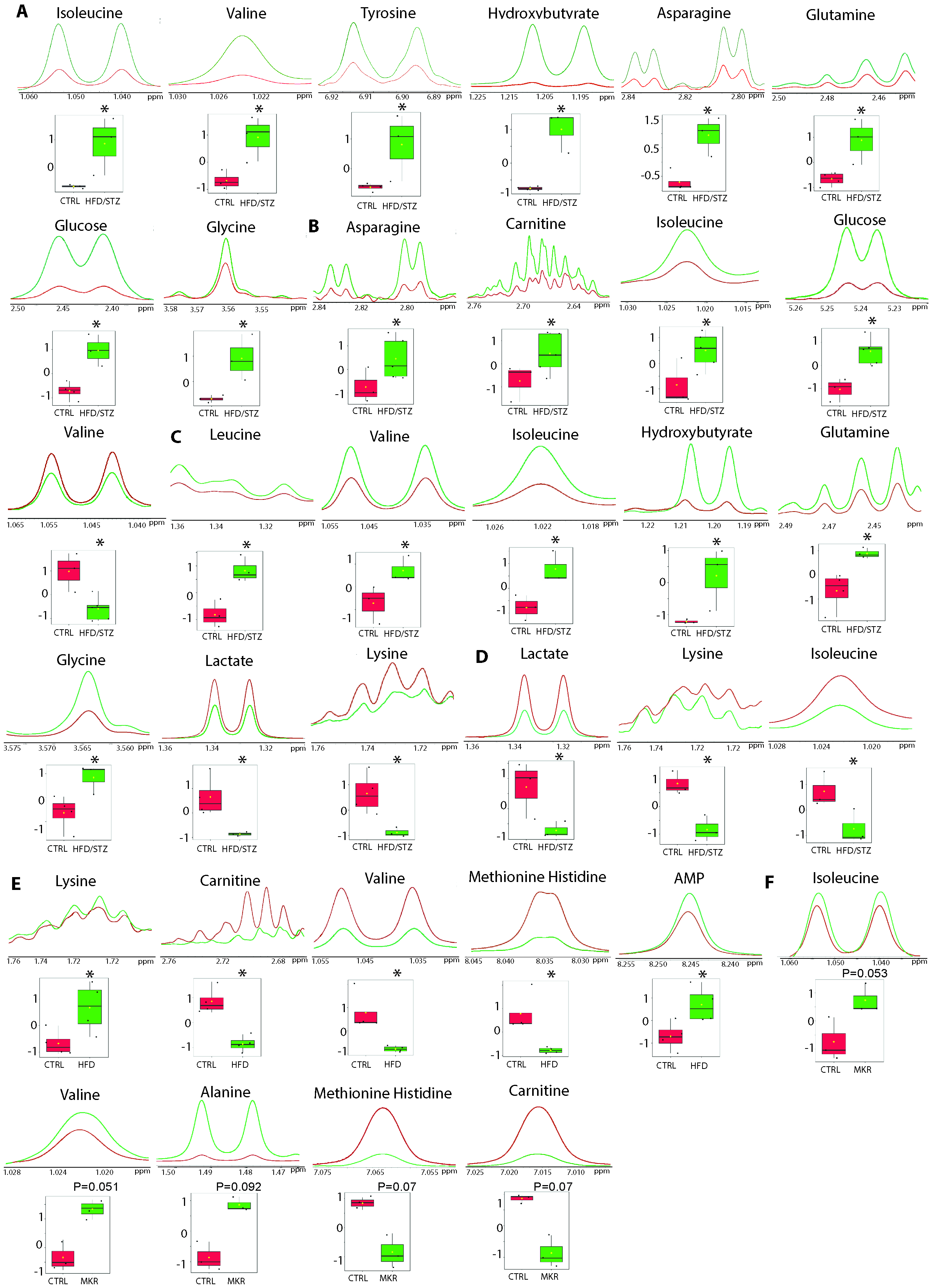

Supplement: Supplementary file 2 — Supplementary Material 2. Supplementary Fig. 2. The metabolomic analysis of quadriceps muscle in the different T2DM mouse models indicated specific metabolic alterations. The evaluation of quadriceps muscle metabolomics at 20 weeks of age by nuclear magnetic resonance spectroscopy (NMR) in male (M) and female (F) HFD/STZ-induced (HFD/STZ) 129/Sv (A, B) and C57 (C, D) mice, HFD-induced (HFD) C57 (E) mice, and MKR male mice from FVB background (F). The diagrams represent the normalized values of diabetic groups in green (HFD/STZ, HFD, MKR) and control groups in red (CTRL), in relation to the centered mean spectral value at 0. The spectral figures represent the relative intensity (y axis) compared to the frequency of resonance of the NMR signal (x axis) of diabetic groups in green (HFD/STZ, HFD, MKR) compared to control groups in red (CTRL). (n = 4 for all groups) All data are shown as mean ± standard deviation. Non-parametric Mann–Whitney test for all genetic backgrounds; * indicate significance (with P < 0.05 *, < 0.005**) relative to controls (CTRL). [file 10020_2024_850_MOESM2_ESM.jpg]

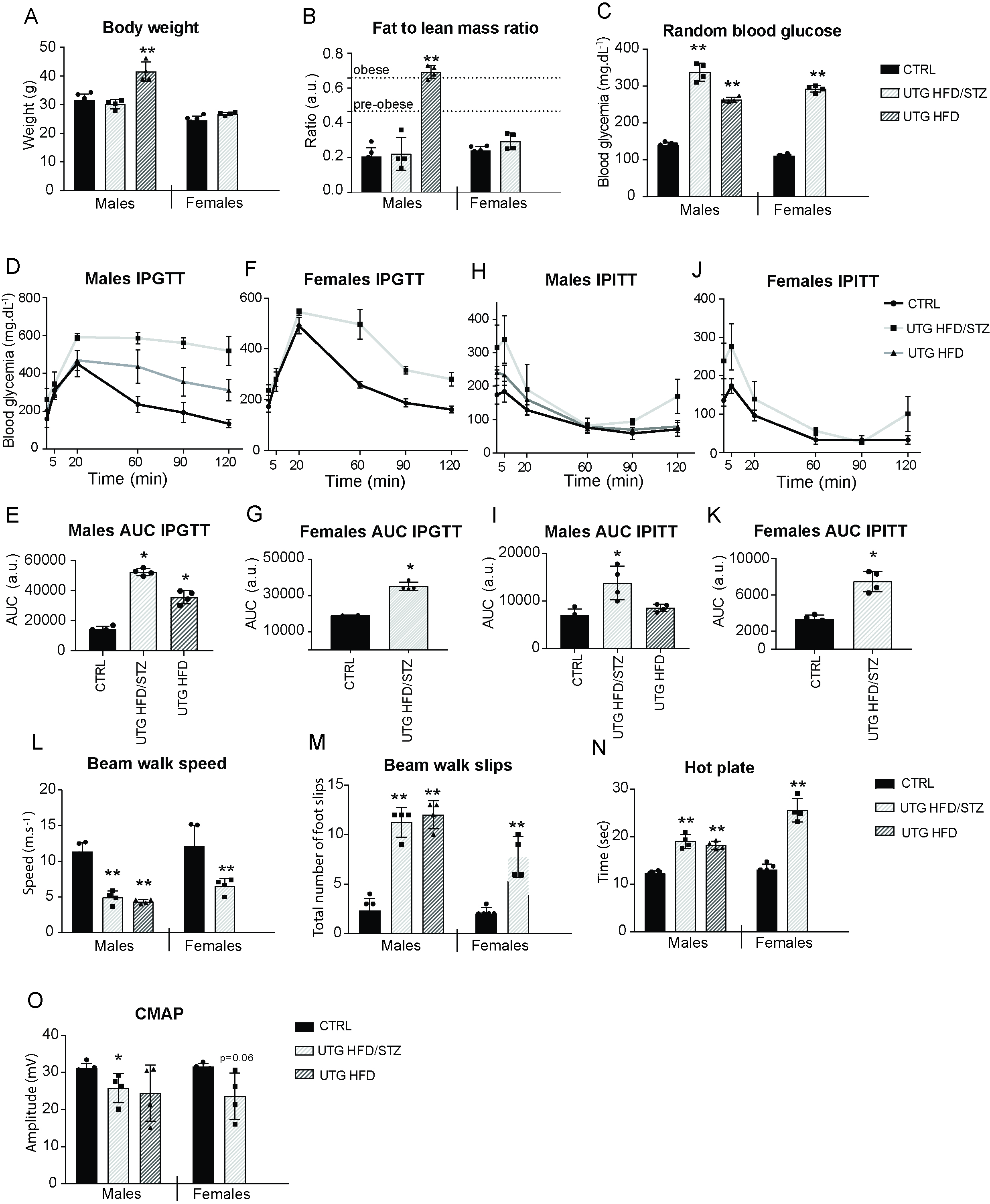

Supplement: Supplementary file 3 — Supplementary Material 3. Supplementary Fig. 3. The sex and method of T2DM induction significantly influence body weight, obesity status, glycemia and sensorimotor function in mice. Body weight assessment (A) was conducted on the untrained groups of male (M) and female (F) mice induced with HFD/STZ (UTG HFD/STZ), as well as HFD-induced (UTG HFD) mice in comparison to CTRL groups. The fat-to-lean mass ratio (B) was calculated using NMR data, with dotted lines indicating the thresholds for determining pre-obese (0.466) or obese (0.658) mice. Non-fasting blood glucose was measured using an Accu-check glucometer (C), and dotted lines indicate the hyperglycemia threshold (250 mg/dl). Glucose tolerance (IPGTT) (D, F) and insulin tolerance (IPITT) (H, J) tests were conducted, and statistical analysis of the area under the curve (AUC) was determined for IPGTT (E, G) and IPITT (I, K). The motor coordination was assessed by i) the beam walk test, during which the speed (L) and foot slips (M) were measured, ii) the thermal sensitivity hot plate test (N) and iii) CMAP amplitude (O) in all untrained groups at 32 weeks of age, compared to controls (CTRL). (n = 5 for all groups). All data are shown as mean ± standard deviation. Non-parametric One-Way ANOVA for all C57 males, and non-parametric Mann–Whitney test for all C57 females; * indicate significance (with P < 0.05 *, < 0.005**) relative to controls (CTRL). [file 10020_2024_850_MOESM3_ESM.jpg]
